# Particulate Matter-Induced Skin Injury: A Dual-Pathway AhR–Nrf2 Framework for Epidermal Homeostasis and Therapeutic Targeting

**DOI:** 10.3390/ijms27177573

**Published:** 2026-08-24

**Authors:** Chia-Hsuan Lin, Chia-Hung Yen, Yu-Tse Wu, Hsun-Shuo Chang, Horng-Huey Ko, Yih-Fung Chen

**Affiliations:** 1 Graduate Institute of Natural Products, College of Pharmacy, Kaohsiung Medical University, Kaohsiung 807378, Taiwan; fros.ta@hotmail.com (C.-H.L.); chyen@kmu.edu.tw (C.-H.Y.); 2 Drug Development and Value Creation Research Center, Kaohsiung Medical University, Kaohsiung 807378, Taiwan; hschang@kmu.edu.tw; 3 Department of Medical Research, Kaohsiung Medical University Hospital, Kaohsiung 807378, Taiwan; ytwu@kmu.edu.tw; 4 School of Pharmacy, College of Pharmacy, Kaohsiung Medical University, Kaohsiung 807378, Taiwan; 5 Center for Cancer Research, Kaohsiung Medical University, Kaohsiung 807378, Taiwan; 6 Department of Fragrance and Cosmetic Science, College of Pharmacy, Kaohsiung Medical University, Kaohsiung 807378, Taiwan; 7 Department of Pharmacy, Kaohsiung Municipal Siaogang Hospital, Kaohsiung 812012, Taiwan; 8 University Center of Bioscience and Biotechnology, National Cheng Kung University, Tainan 70101, Taiwan

**Keywords:** aryl hydrocarbon receptor (AhR), dual activation, natural products, nuclear factor erythroid 2-related factor 2 (Nrf2), particulate matter (PM), oxidative stress, skin barrier function

## Abstract

The aryl hydrocarbon receptor (AhR) is highly expressed in keratinocytes and functions as an environmental sensor regulating xenobiotic metabolism, epidermal differentiation, and inflammatory responses. Particulate matter (PM), a major environmental pollutant containing reactive oxygen species (ROS), transition metals, and polycyclic aromatic hydrocarbons (PAHs), induces oxidative stress and inflammation, leading to skin barrier dysfunction. Transition metals generate ROS via Fenton-type reactions, whereas PAHs undergo AhR-mediated metabolism that further amplifies oxidative stress. Excessive ROS promotes inflammatory cytokine expression and disrupts barrier-related protein expression. In response, activation of the nuclear factor erythroid 2-related factor 2 (Nrf2) pathway induces antioxidant enzymes, including heme oxygenase-1 (HO-1), to counteract oxidative damage. However, sustained PM exposure may overwhelm these defense mechanisms, resulting in impaired cellular homeostasis. Although the roles of AhR and Nrf2 have been extensively investigated individually, their coordinated regulation in PM-induced skin injury remains underexplored. This review summarizes current evidence on the functional interplay between AhR and Nrf2 and discusses how coordinated activation of these pathways integrates xenobiotic metabolism, antioxidant defense, and barrier-associated functions. Overall, the available evidence supports a dual-pathway framework for maintaining epidermal homeostasis under PM-induced environmental stress.

## 1. Introduction

### 1.1. Environmental Particulate Matter as an Emerging Public Health Concern

Particulate matter (PM) is a ubiquitous air pollutant with significant adverse health outcomes [1,2]. According to the United States Environmental Protection Agency (US EPA), PM is classified by aerodynamic diameter into coarse particles (PM10, <10 μm), fine particles (PM2.5, <2.5 μm), and ultrafine particles (UFPs, <100 nm) [3,4]. PM originates from multiple anthropogenic and natural sources, including traffic emissions, industrial combustion, fossil fuel burning, wildfire smoke, and secondary atmospheric reactions [5]. The relative contributions of these sources determine particle size distribution and chemical composition, thereby influencing biological reactivity.

Particle size critically affects deposition behavior, cellular interaction, and toxicological outcomes [6]. As aerodynamic diameter decreases, particles exhibit increased surface area relative to mass and enhanced ability to penetrate biological barriers [7]. This amplified surface area-to-mass ratio increases surface reactivity, allowing finer particles to adsorb higher concentrations of toxic trace elements and organic compounds [8,9]. PM2.5 and UFPs exhibit prolonged atmospheric suspension and deeper tissue penetration compared with coarse particles [4,9,10]. Recent evidence suggests a negative association between particle size and in vitro toxicity, with PM2.5 fractions inducing significantly stronger inflammatory reactions in macrophages than coarser particles [11,12].

Beyond its physical dimensions, the toxicity of PM is fundamentally dictated by its highly heterogeneous chemical payload and the resulting physicochemical properties, particularly surface charge and hydrophobicity [13]. PM contains transition metals (e.g., Fe, Mn, Mo, and Cu), polycyclic aromatic hydrocarbons (PAHs), and other redox-active constituents [14,15,16]. Surface-bound reactive species possess redox activity and can induce oxidative reactions in exposed tissues [15]. Water-soluble fractions enriched in bioavailable transition metals catalyze Fenton-type reactions, facilitating the conversion of hydrogen peroxide into hydroxyl radicals [17]. Meanwhile, hydrophobic organic fractions enriched in PAHs, such as benzo[a]pyrene, readily partition into lipid membranes and enter cells [18,19]. Together, these physicochemical characteristics contribute to oxidative stress-related health effects.

Due to their small size and surface reactivity, PM2.5 and UFPs can bypass primary clearance mechanisms and enter the body through inhalation, ingestion, and dermal exposure [6]. Inhaled particles may penetrate the alveolar region and translocate into systemic circulation [9]. Following dermal exposure, particularly when the skin barrier is compromised, nanoscale particles and associated toxicants may penetrate beyond the stratum corneum and affect viable epidermal cells [20]. Epidemiological data from the World Health Organization (WHO) associate long-term PM exposure with respiratory diseases, cardiovascular disorders, metabolic dysfunction, and increased mortality [21,22,23]. Emerging evidence from epidemiological studies and mechanistic investigations further links chronic PM exposure to accelerated skin aging and exacerbation of inflammatory skin diseases, including atopic dermatitis [24,25,26].

Unlike ultraviolet (UV) radiation, which primarily induces photochemical DNA damage [27], or ozone, which reacts predominantly at the skin surface due to limited penetration [28], PM acts as both a particulate carrier and a source of intracellularly active chemical constituents [29]. The combination of physical deposition and metabolic activation distinguishes PM-induced cutaneous injury from other environmental stressors. Understanding the health consequences of PM exposure is therefore essential for developing preventive and therapeutic strategies. However, accurately characterizing PM-induced cellular responses remains technically challenging, partly due to the complex physicochemical properties of PM and the intrinsic autofluorescence of PAHs-enriched organic fractions, which can interfere with fluorescence-based analyses [30,31]. These limitations underscore the need for advanced analytical approaches capable of resolving the spatial and phenotypic heterogeneity of PM-induced skin injury. In this context, a previously established imaging platform provides a useful tool for visualizing PM-induced cellular responses and supporting the mechanistic interpretation of skin barrier dysfunction [32].

### 1.2. The Skin as the Primary Defense Interface Against Environmental Pollutants

As the primary interface between the human body and the external environment, the skin’s structural integrity is crucial for defense [33,34]. Structurally, the cutaneous barrier relies on two functional layers: the supportive dermis and the highly stratified epidermis [35]. The dermis consists of a collagen- and elastin-rich extracellular matrix that provides tensile strength and mechanical support [36]. The epidermis, the outermost layer of the skin, serves as the primary physical, chemical, and immunological interface with the external environment [37]. It is a stratified, avascular epithelium composed predominantly of keratinocytes, which undergo a tightly regulated differentiation program from the stratum basale to the outermost stratum corneum [38].

At the terminal stage of this differentiation process, the stratum corneum, approximately 10–20 μm thick in humans, is formed by terminally differentiated, anucleated corneocytes embedded within a hydrophobic intercellular lipid matrix [39]. This highly compact organization establishes the principal permeability barrier of the skin. Each corneocyte is surrounded by a highly cross-linked proteinaceous cornified envelope composed of filaggrin, loricrin, involucrin, and small proline-rich proteins, conferring mechanical strength and contributing to hydration homeostasis [40,41]. In the viable epidermis, keratinocytes are interconnected by specialized cell–cell junctions, including tight junctions and adherens junctions, which further restrict paracellular diffusion of water, ions, and xenobiotics. These junctions contain key components such as zonula occludens-1 (ZO-1) and E-cadherin/β-catenin complexes, respectively [42,43,44]. Additionally, integral membrane proteins such as aquaporin-3 (AQP3) facilitate the transport of water and glycerol, serving as a crucial molecular determinant of epidermal hydration and barrier resilience [45,46,47].

Despite its barrier organization, airborne PM continuously interacts with the skin’s surface through adhesion and deposition [48,49]. Although the macroscopic surface area of adult human skin is estimated at approximately 1.8–2.0 m^2^, the presence of hair follicles and sweat ducts increases the effective interactive surface to approximately 25–30 m^2^ [34]. In this structural context, PM may traverse the cutaneous barrier via three principal direct pathways: transcellular, intercellular, and trans-appendageal routes [20,50]. The transcellular route involves diffusion across corneocytes and their cornified envelopes and is generally associated with small, relatively hydrophilic molecules. In contrast, the intercellular route involves diffusion through the lipid matrix between corneocytes and is generally associated with lipophilic compounds. The trans-appendageal route utilizes hair follicles and sweat gland ducts, which serve as preferential deposition sites and reservoirs for particles.

Nanoparticles in the ~40 nm size range exhibit enhanced follicular accumulation and may access perifollicular tissue under barrier-disrupted conditions, whereas larger particles predominantly remain confined within the follicular duct [49,51]. Given that UFPs overlap with this size range, the nanoscale fraction of PM may exhibit increased potential for appendageal deposition and penetration, especially in compromised skin. In addition to particle size, the physicochemical properties of PM-associated chemicals critically influence their route of entry. Lipophilic organic constituents, including PAHs, readily partition into the lipophilic intercellular lipid matrix of the stratum corneum, where highly lipophilic congeners tend to form a superficial reservoir rather than efficiently permeating into deeper viable layers [52]. Together, these structural and compositional determinants position the epidermis as a dynamic interface where particulate pollutants may deposit, persist, and interact with viable cellular compartments.

Functionally, the skin is not merely a passive physical shield but also a metabolically active organ [53]. Keratinocytes express xenobiotic-metabolizing enzymes, including extrahepatic cytochrome P450 isoforms, enabling local biotransformation of environmental pollutants [54]. Consequently, PM-associated organic constituents, such as PAHs, may undergo metabolic activation within the epidermis, generating reactive intermediates that amplify oxidative stress and inflammatory signaling. Collectively, the skin’s complex architecture, extensive environmental exposure, and metabolic responsiveness render it highly susceptible to particulate pollutants.

### 1.3. Mechanistic Overview of PM-Induced Skin Damage

Following cutaneous deposition and epidermal penetration, PM initiates a coordinated cascade of pathogenic events within the viable epidermis [55]. While the physical dimensions of PM determine epidermal penetration depth, its complex chemical payload, comprising transition metals, PAHs, and other redox-active constituents, directly interacts with keratinocytes and drives cytotoxicity [56]. Building upon the physicochemical properties described earlier, these internalized constituents trigger excessive reactive oxygen species (ROS) generation through distinct cellular mechanisms: surface-bound constituents directly induce local oxidative reactions, intracellular transition metals rapidly catalyze Fenton-type reactions, and PAHs strongly activate the cytosolic aryl hydrocarbon receptor (AhR), which transcriptionally induces cytochrome P450 enzymes (e.g., CYP1A1), leading to the formation of reactive electrophilic intermediates and secondary ROS [57]. Collectively, this excessive ROS generation establishes oxidative stress as the central pathogenic driver of PM-induced epidermal injury.

The accumulation of ROS disrupts cellular redox homeostasis, leading to lipid peroxidation, protein oxidation, mitochondrial dysfunction, and DNA damage [58]. Concurrently, oxidative stress activates redox-sensitive signaling pathways, including mitogen-activated protein kinases (MAPKs) and nuclear factor kappa-light-chain-enhancer of activated B cells (NF-κB) cascades, which amplify pro-inflammatory cytokine production and promote immune cell recruitment [59]. In response, endogenous antioxidant defense mechanisms are engaged, primarily through activation of the nuclear factor erythroid 2–related factor 2 (Nrf2) signaling pathway, which coordinates transcriptional cytoprotective programs [60]. However, when ROS generation overwhelms cellular antioxidant defenses, persistent oxidative stress results in keratinocyte cytotoxicity, apoptosis, and amplified inflammatory responses that progressively compromise epidermal integrity, ultimately exacerbating cutaneous inflammatory disorders such as atopic dermatitis [25,56].

**Figure 1:**
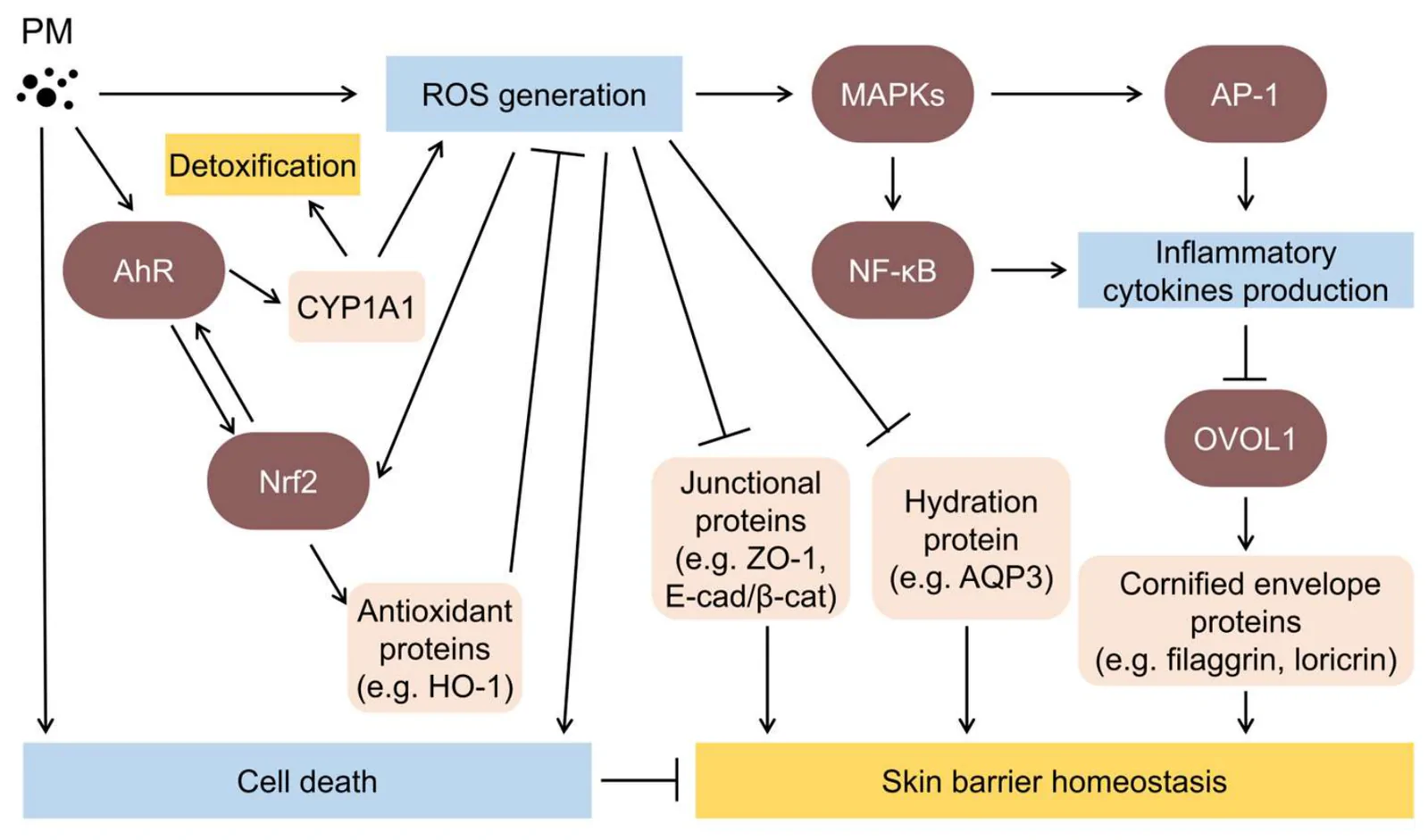
Environmental PM induces epidermal damage. Environmental PM disrupts epidermal homeostasis through multiple interconnected signaling pathways. PM-associated PAHs activate AhR signaling, which exerts dual effects: on one hand, AhR activation enhances xenobiotic metabolism via CYP1A1, leading to increased generation of ROS; on the other hand, it can induce Nrf2 activation and downstream antioxidant responses, including HO-1, as a compensatory mechanism. Excessive ROS further amplifies oxidative stress while activating MAPK signaling, which in turn stimulates transcription factors such as AP-1 and NF-κB, promoting inflammatory cytokine production. These inflammatory pathways suppress OVOL1-mediated epidermal differentiation and impair the formation of cornified envelope proteins. Concurrently, ROS disrupts epidermal barrier integrity by downregulating junctional proteins and reducing hydration-associated proteins such as AQP3. Collectively, these processes lead to keratinocyte damage, impaired barrier homeostasis, and ultimately cell death under PM exposure.

Barrier dysfunction represents a critical pathological hallmark of PM exposure [61]. Mechanistically, in vitro studies using human epithelial models have shown that PM exposure reduces and delocalizes essential structural proteins involved in barrier formation, including filaggrin, loricrin, and involucrin, while simultaneously disrupting tight junction and adherens junction components [62,63,64]. Oxidative damage further impairs hydration-related molecules such as AQP3 and delays barrier recovery [65]. Notably, beyond its role in xenobiotic metabolism and ROS amplification, AhR also functions as a context-dependent regulator of epidermal differentiation and barrier homeostasis [66,67]. Consequently, epidermal barrier integrity under PM exposure depends on the dynamic balance between sustained structural injury and context-dependent regulatory responses mediated by pathways such as AhR. To elucidate these complex interactions, the following sections describe the oxidative mechanisms underlying PM-induced injury, the resulting cellular consequences, the disruption of epidermal barrier integrity, and the regulatory roles of AhR and Nrf2 signaling (Figure 1).

## 2. Molecular Pathways Regulating Cellular Responses to PM Exposure

### 2.1. Oxidative Stress-Driven Mechanisms Underlying PM-Induced Skin Injury

At the cellular level, the detrimental effects of PM are largely mediated by excessive ROS production, which disrupts intracellular redox homeostasis and is a central driver of pollution-induced skin injury [55,59]. PM-associated oxidative stress involves multiple ROS with distinct chemical reactivities, including superoxide anion (O2^•–^), hydrogen peroxide (H_2_O_2_), and the highly reactive hydroxyl radical (^•^OH) [17,68]. Initial physicochemical interactions between PM constituents and cellular components commonly generate superoxide anion, which is rapidly converted to hydrogen peroxide by intracellular superoxide dismutase (SOD) [69]. A critical amplification step occurs when hydrogen peroxide encounters bioavailable transition metals derived from PM [70]. Through classical Fenton and Haber–Weiss reactions, these metals catalyze the conversion of hydrogen peroxide into highly reactive hydroxyl radicals, thereby markedly amplifying intracellular oxidative stress [71].

In addition to these direct physicochemical reactions, PM exposure further amplifies oxidative stress through endogenous cellular sources [72]. PM has been reported to impair mitochondrial electron transport chain function, resulting in electron leakage and increased production of mitochondrial ROS [70]. Furthermore, PM constituents can activate innate immune signaling pathways. For example, studies using human keratinocytes and murine skin models have demonstrated that direct interaction between toll-like receptor 5 (TLR5) and NADPH oxidase 4 (NOX4) has been reported to sustain intracellular ROS generation following PM exposure [73]. These combined processes create a persistent oxidative microenvironment that extends beyond the initial physicochemical insult.

Excessive ROS accumulation subsequently induces oxidative damage to multiple cellular macromolecules. Lipid membranes, particularly those enriched in polyunsaturated fatty acids, are highly susceptible to ROS-mediated lipid peroxidation, resulting in the formation of reactive aldehydes such as malondialdehyde (MDA) and 4-hydroxynonenal (4-HNE) [74,75]. Proteins are similarly vulnerable to oxidative modification, including carbonylation and nitration, which impair the function of structural and enzymatic proteins [76]. In addition, ROS-mediated DNA damage, exemplified by oxidative lesions such as 8-oxo-2′-deoxyguanosine (8-oxo-dG), contributes to genomic instability and mutagenesis [77,78,79]. These oxidative modifications collectively compromise cellular integrity and trigger stress responses in epidermal cells.

Beyond direct molecular damage, ROS also function as key signaling mediators that activate multiple redox-sensitive pathways. Elevated oxidative stress stimulates MAPK cascades, including ERK1/2, JNK, and p38, as well as downstream transcription factors such as NF-κB and activator protein-1 (AP-1) [55]. Activation of these pathways promotes the expression of numerous pro-inflammatory mediators, including tumor necrosis factor-α (TNF-α), interleukin-1β (IL-1β), interleukin-6 (IL-6), cyclooxygenase-2 (COX-2), and matrix metalloproteinases (MMPs), thereby linking oxidative stress to inflammatory responses and extracellular matrix degradation in the skin [56].

Nevertheless, when PM-induced ROS generation exceeds the protective capacity of these antioxidant systems, persistent oxidative stress promotes keratinocyte dysfunction, apoptosis, and sustained inflammatory signaling. Collectively, these oxidative processes represent a central mechanistic link between PM exposure and the subsequent development of cellular injury and epidermal barrier disruption.

### 2.2. Cellular and Molecular Consequences of PM Exposure

Beyond the disruption of cellular redox homeostasis described in Section 2.1, PM-induced oxidative stress exerts direct effects on intracellular organelles within keratinocytes. Following cellular uptake, PM particles have been observed predominantly in secondary lysosomes, autophagy vesicles, and endocytotic vesicles in cultured primary keratinocytes [49]. This intracellular localization may facilitate interactions between PM-associated constituents and cellular components, contributing to oxidative stress, organelle dysfunction, and downstream cytotoxic responses. Mitochondrial dysfunction can further interfere with electron transport chain activity, leading to mitochondrial membrane depolarization and impaired ATP production [80]. This metabolic impairment not only compromises cellular energy metabolism but also sensitizes keratinocytes to subsequent stress signals.

In parallel with organelle impairment, PM exposure also induces substantial genomic damage. PM constituents, particularly PAHs and transition metals, possess genotoxic properties that promote the formation of DNA single- and double-strand breaks [63,81]. In response to such genomic stress, keratinocytes activate cell-cycle checkpoint mechanisms to prevent the propagation of damaged DNA. Several studies have reported that PM exposure induces cell-cycle arrest in keratinocytes, most commonly at the G0/G1 phase [82,83,84]. However, the precise arrest phase may vary depending on the physicochemical characteristics of PM and the cellular context. For example, toxicological studies in respiratory epithelia models, such as BEAS-2B cells, have reported delayed S-phase progression, G2/M arrest, or complete mitotic arrest following PM exposure [85]. These variations highlight the influence of particle composition, oxidative burden, and cell types on the PM-induced cellular stress response.

The accumulation of organelle dysfunction and genomic instability ultimately results in cytotoxicity. Numerous in vitro studies indicate that PM exposure leads to dose- and time-dependent reductions in keratinocyte viability, often accompanied by biochemical indicators of cytotoxicity such as increased lactate dehydrogenase (LDH) release [86,87]. Such cellular deterioration reflects severe impairment of metabolic activity and membrane integrity following PM exposure, ultimately leading to programmed cell death. In HaCaT keratinocytes, PM exposure alters the balance of Bcl-2 family proteins by downregulating the anti-apoptotic protein Bcl-2 and upregulating pro-apoptotic factors such as Bax, thereby promoting mitochondrial outer membrane permeabilization and the subsequent release of cytochrome c into the cytosol [88]. This mitochondrial signaling event activates the caspase cascade, particularly caspase-9, followed by the cleavage of caspase-3, ultimately leading to the cleavage of poly(ADP-ribose) polymerase (PARP) [87,89,90]. Collectively, these events characterize the intrinsic mitochondrial apoptotic pathway triggered by PM exposure.

While apoptosis represents the predominant form of PM-induced cell death, excessive cellular injury may shift the cellular response toward necrotic damage. Cellular energy depletion, damage to membrane lipids, and the loss of function of homeostatic ion pumps/channels can synergistically induce necrosis [91]. Experimental models have reported increased propidium iodide uptake in PM-exposed keratinocytes, suggesting membrane damage consistent with necrotic injury [58]. Crucially, unlike apoptosis, the necrotic rupture of keratinocytes releases intracellular contents and lysosomal hydrolases into the surrounding microenvironment [92]. This uncontrolled exudation acts as a potent pro-inflammatory trigger, exacerbating local tissue damage and playing a pivotal role in the severe epidermal barrier dysfunction and chronic inflammation associated with pollution exposure.

In addition to apoptosis and necrosis, other forms of cell death, such as autophagy-associated cell death, have also been reported following PM exposure [91]. As a lysosome-dependent degradation pathway responsible for removing damaged organelles and protein aggregates, autophagy may initially function as a protective mechanism to mitigate cellular injury. This is experimentally monitored through the lipidation of LC3 (conversion from LC3-I to LC3-II) and the degradation of the cargo receptor p62 [86,93]. However, prolonged or dysregulated autophagy under severe PM exposure can transition from cytoprotective to cytotoxic, highlighting the complex interplay between adaptive and destructive cellular responses.

In addition to acute cytotoxicity and cell death, prolonged PM exposure in cellular models induces persistent ROS-dependent DNA damage, driving keratinocytes and dermal fibroblasts into irreversible cell-cycle arrest and premature cellular senescence [94,95,96]. Persistent oxidative stress activates senescence-related pathways, including p16^INK4a^, p21, and p53 signaling, leading to the accumulation of senescence-associated β-galactosidase (SA-β-gal) activity and the loss of lamin B1 [97,98]. Consequently, these senescent skin cells develop a senescence-associated secretory phenotype (SASP), which is characterized by the sustained release of pro-inflammatory cytokines, thereby amplifying local epidermal inflammation [99,100]. In parallel, the SASP-driven secretion of MMPs (e.g., MMP-1, MMP-3, and MMP-9) accelerates the degradation of collagen I and elastin, resulting in reduced dermal structural integrity, loss of skin elasticity, and accelerated premature skin aging [101,102].

PM exposure is also closely associated with hyperpigmentation through stress-induced keratinocyte–melanocyte crosstalk [103]. Beyond matrix degradation, the dysfunctional secretory activity of senescent keratinocytes serves as a potent paracrine trigger for melanogenesis [104]. Through the release of SASP mediators, PM-stressed keratinocytes aberrantly stimulate neighboring melanocytes and promote melanin synthesis [103,105]. Consistent with this mechanism, PM exposure has been associated with the upregulation of key melanogenic regulators, including melanocyte-inducing transcription factor (MITF), tyrosinase (TYR), and tyrosinase-related proteins (TRP-1 and TRP-2) [106]. Although the extent of pigmentation changes may vary depending on intrinsic individual susceptibility [107], chronic pollution exposure is commonly associated with hyperpigmentation disorders and uneven skin tone.

Collectively, these cellular and molecular consequences of PM exposure, encompassing organelle dysfunction, genomic instability, cytotoxicity, programmed cell death, cellular senescence, and premature aging-associated alterations, substantially impair overall epidermal homeostasis. The resulting loss of functional keratinocytes, together with extracellular matrix degradation and persistent inflammatory amplification, weakens epidermal structural resilience and promotes the development of pollution-associated skin barrier dysfunction.

### 2.3. PM-Induced Disruption of Epidermal Barrier Integrity

Keratinocytes constitute the primary cellular component of the epidermis and are responsible for producing structural proteins and maintaining intercellular junctions. Their dysfunction can therefore compromise epidermal cohesion and barrier integrity [56].

PM exposure has been reported to disrupt the expression of key cornified envelope proteins that are essential for epidermal cohesion and differentiation. For example, exposure to the urban particulate standard reference material SRM 1649b significantly reduced the mRNA expression of filaggrin, involucrin, and loricrin in mouse skin [108]. Similarly, PM2.5 exposure suppresses filaggrin gene expression in cultured keratinocytes and induces abnormal protein localization in organotypic skin models, accompanied by increased transepidermal water loss (TEWL), indicating impaired barrier function [109]. These observations suggest that PM interferes with keratinocyte differentiation and cornified envelope formation, thereby weakening epidermal cohesion.

In addition to altering the expression and localization of cornified envelope proteins, PM exposure disrupts intercellular junction complexes that regulate epidermal permeability. Experimental studies in human keratinocytes, corneal epithelial cells, and murine skin indicate that PM exposure and the associated oxidative stress promote the degradation and cytoplasmic redistribution of the tight junction protein ZO-1, leading to compromised junctional assembly and increased epithelial permeability [62,63,108]. Disruption of adherens junction components may further exacerbate epidermal instability. In an ex vivo pig skin model, PM exposure reduced E-cadherin expression and induced its abnormal redistribution toward the lower layers of the epidermis, thereby weakening intercellular adhesion [90]. Desmosomal components may also be affected by PM exposure. In a human three-dimensional skin model, PM exposure inhibited the gene expression of desmocollin-1 and corneodesmosin, which contribute to intercellular adhesion in the epidermis [109].

PM exposure also interferes with mechanisms that regulate epidermal hydration. Experimental evidence indicates that PM-induced oxidative stress can disturb AQP3-mediated water transport in keratinocytes, thereby contributing to epidermal dehydration and impaired barrier repair [65,110,111]. Consistent with the importance of AQP3 in epidermal homeostasis, dysregulation of this protein has been implicated in multiple inflammatory skin disorders, including atopic dermatitis, psoriasis, vitiligo, and chronic pruritus [45,112].

Collectively, PM exposure disrupts epidermal barrier integrity through multiple complementary mechanisms, including impairment of cornified envelope proteins, destabilization of cell–cell junctions, and dysregulation of hydration-regulating pathways. These alterations weaken epidermal cohesion and increase transepidermal permeability, thereby facilitating the development and progression of pollution-associated skin disorders.

Beyond barrier impairment, PM-induced skin injury involves a broader network of interconnected biological responses, ranging from early xenobiotic sensing and oxidative stress to mitochondrial dysfunction, cell fate dysregulation, inflammatory amplification, and tissue-level pathological alterations. These events collectively impair epidermal homeostasis and contribute to pollution-associated skin disorders. A systematic summary of the major biological responses and tissue-level pathological outcomes associated with PM-induced skin damage, together with representative molecular targets and experimental indicators, is presented in Table 1.

### 2.4. Context-Dependent Roles of the AhR in Skin Barrier Regulation

The AhR is widely expressed in major skin cell types and functions as a ligand-activated transcription factor that senses a wide spectrum of environmental chemicals, dietary metabolites, and endogenous signaling molecules [66]. As an environmental sensor, AhR plays essential roles in xenobiotic metabolism, immune regulation, and epidermal differentiation [113]. In the skin, AhR signaling is increasingly recognized as a key molecular interface linking environmental exposures with epidermal barrier regulation [114,115].

In the canonical AhR signaling pathway, AhR resides in the cytoplasm in its inactive state as part of a multiprotein chaperone complex consisting of 90 kDa heat shock protein (HSP90), AhR-interacting protein (AIP), p23, and the tyrosine kinase c-SRC. Upon ligand binding, AhR undergoes conformational activation and translocates into the nucleus, where it heterodimerizes with the AhR nuclear translocator (ARNT). The resulting AhR–ARNT complex binds xenobiotic response elements (XREs) in target gene promoters and initiates transcription of canonical detoxification genes, most prominently cytochrome P450 enzymes such as CYP1A1 [115,116,117,118]. In addition to cytochrome P450 enzymes, AhR activation also induces the AhR repressor (AhRR), which forms a negative feedback loop that limits excessive AhR signaling by competing with AhR for ARNT binding [119]. 

**Table 1:** Major biological responses and pathological outcomes associated with PM-induced skin damage.

Key Events	Molecular Targets/Biomarkers	References
**Upstream stress sensing & xenobiotic response**		
ROS generation & stress signaling	TLR5 ↑, NOX4 ↑, ROS ↑, MAPKs activation, NF-κB/AP-1 activation	[73,120,121,122]
Xenobiotic metabolism	AhR nuclear translocation, CYP1A1 ↑, CYP1B1 ↑, AhRR ↑	[32,63,122]
Keap1–Nrf2 antioxidant response	Nrf2 nuclear translocation, Nrf2 ↑, HO-1 ↑	[32,123,124,125]
**Oxidative stress & molecular damage**		
Redox imbalance	GSH ↓, GSH-Px ↓, GSTs ↑, SOD ↓, CAT ↓, GCLC ↓	[100,123,124,126]
Lipid peroxidation & protein oxidation	Lipid oxidative stress DPPP ↑, protein carbonylation, MDA ↑, 4-HNE ↑	[58,75,87,121,124,126,127,128]
DNA damage response	γH2AX ↑, DNA fragmentation, DNA tail ↑, 8-oxo-dG ↑, p53 activation	[58,63,80,121,126,129]
**Organelle dysfunction**		
Mitochondrial dysfunction	ATP ↓, Δψ_m_ collapse, cytochrome c release	[80,121,124,126,130]
Autophagy–lysosomal dysfunction	LC3-II/LC3-I ratio ↑, p62/SQSTM1 ↓	[86,93,121,129]
Plasma membrane damage	LDH release	[86,87,127]
**Cell fate regulation**		
Cell-cycle arrest	G0/G1 arrest, Sub-G1 ↑, p16 ↑, p21 ↑, p27 ↑, Cyclin D1 ↓, Cyclin E ↓, CDK2 ↓, CDK4 ↓	[80,84,121,129]
Programmed cell death	Bax ↑, Bcl-2 ↓, cytochrome c ↑, caspase-9/3 cleavage ↑, PARP cleavage ↑, apoptotic bodies ↑	[80,87,90,121,124,126]
Senescence	p16 ↑, SA-β-gal ↑	[82,94]
**Tissue-level pathological outcomes**		
Inflammation	NF-κB/AP-1 nuclear translocation, NLRP1 inflammasome, IL-1β ↑, IL-6 ↑, TNF-α ↑, COX-2 ↑, iNOS ↑	[65,122,127,128,131,132,133]
Skin barrier dysfunction	Tight junctions: ZO-1 ↓, claudin-1 ↓, occludin ↓ Adherens junctions: E-cadherin ↓, β-catenin ↓ Desmosomal components: desmocollin-1 ↓, corneodesmosin ↓ Cornified envelope: FLG ↓, IVL ↓, LOR ↓	[32,63,64,65,90,108,109,134]
Skin hydration imbalance	TEWL ↑, AQP3 ↓, NMF ↓	[65,90,109,135]
Melanogenesis	MITF ↑, TYR ↑, TRP-1 ↑, TRP-2 ↑	[103,105]
Premature aging	MMP-1 ↑, MMP-3 ↑, MMP-9 ↑, collagen I ↓, elastin ↓	[82,103,128,136]

Abbreviation: AP-1, activator protein-1; ATP, adenosine triphosphate; AQP3, aquaporin-3; AhR, aryl hydrocarbon receptor; AhRR, AhR repressor; CAT, catalase; COX-2, cyclooxygenase-2; CYP1A1, cytochrome P450 1A1; DPPP, diphenyl-1-pyrenylphosphine; FLG, filaggrin; GCLC, glutamate–cysteine ligase catalytic subunit; GSH, glutathione; GSH-Px, glutathione peroxidase; GSTs, glutathione S-transferases; HO-1, heme oxygenase 1; 4-HNE, 4-hydroxynonenal; iNOS, inducible nitric oxide synthase; IL-1β, interleukin-1β; IVL, involucrin; LDH, lactate dehydrogenase; LOR, loricrin; MDA, malondialdehyde; MMP-1, matrix metalloproteinases-1; MITF, melanocyte-inducing transcription factor; LC3-I, cytosolic form of microtubule-associated protein 1 light chain 3; LC3-II, lipidated form of microtubule-associated protein 1 light chain 3; Δψm, mitochondrial membrane potential; MAPKs, mitogen-activated protein kinase; NOX4, NADPH oxidase 4; NMF, natural moisturizing factor; NLRP1, NLR family pyrin domain containing 1; Nrf2, nuclear factor erythroid 2-related factor 2; NF-κB, nuclear factor kappa-light-chain-enhancer of activated B cells; 8-oxo-dG, 8-oxo-2′-deoxyguanosine; γH2AX, phosphorylated histone H2AX; PARP, poly(ADP-ribose) polymerase; ROS, reactive oxygen species; SA-β-gal, senescence-associated β-galactosidase; SOD, superoxide dismutase; TLR5, toll-like receptor 5; TEWL, transepidermal water loss; TNF-α, tumor necrosis factor-α; TYR, tyrosinase; TRP-1, tyrosinase-related protein 1; ZO-1, zonula occludens-1. ↑. Increase; ↓, Decrease.

Beyond this classical pathway, AhR also participates in non-canonical signaling networks through interactions with other transcriptional regulators, including NF-κB, thereby modulating inflammatory gene expression [137,138]. In addition, AhR has been reported to function as an E3 ubiquitin ligase capable of promoting the proteasomal degradation of specific protein targets [139], underscoring its role as a multifunctional signaling protein.

Physiologically, AhR signaling contributes to the maintenance of epidermal barrier integrity [140]. Activation of AhR induces the transcription factor ovo-like 1 (OVOL1), forming the AhR–OVOL1–filaggrin regulatory axis that drives keratinocyte differentiation and terminal epidermal maturation [141,142,143]. In parallel, AhR signaling has been reported to regulate the expression of several junctional proteins involved in epithelial barrier function, including claudin-4, occludin, and the scaffold protein ZO-1, thereby strengthening paracellular barrier integrity [144,145,146]. Collectively, these observations indicate that appropriate AhR activation supports keratinocyte differentiation and maintains structural epidermal barrier stability.

Despite these protective physiological roles, AhR signaling in the skin exhibits a highly context-dependent nature that has been described as “Janus-faced” [147]. The biological outcome of AhR activation is largely determined by the chemical properties, metabolic stability, and persistence of the activating ligand, whether derived from environmental toxins, dietary phytochemicals, microbial metabolites, or pharmaceutical compounds [113]. Many endogenous or dietary ligands, such as the tryptophan photoproduct 6-formylindolo [3,2-*b*]carbazole (FICZ), are rapidly metabolized by CYP1A1 enzymes induced by AhR activation [148]. This rapid metabolic clearance generates a transient activation cycle that limits prolonged receptor signaling and supports physiological processes such as epithelial barrier maintenance and controlled immune responses [149,150].

In contrast, numerous environmental toxicants, including PAHs associated with PM, act as persistent AhR ligands that resist efficient metabolic clearance [151]. These lipophilic compounds bind AhR with high affinity and drive sustained CYP1A1 expression. During CYP1A1-mediated metabolism, PAHs can be converted into highly reactive intermediates, including redox-active quinones, which undergo repeated redox cycling and generate excessive ROS [54,57]. The persistence of PAHs therefore results in prolonged AhR activation and continuous oxidative stress, shifting AhR signaling away from physiological barrier regulation toward cytotoxic and inflammatory outcomes [152,153].

**Figure 2:**
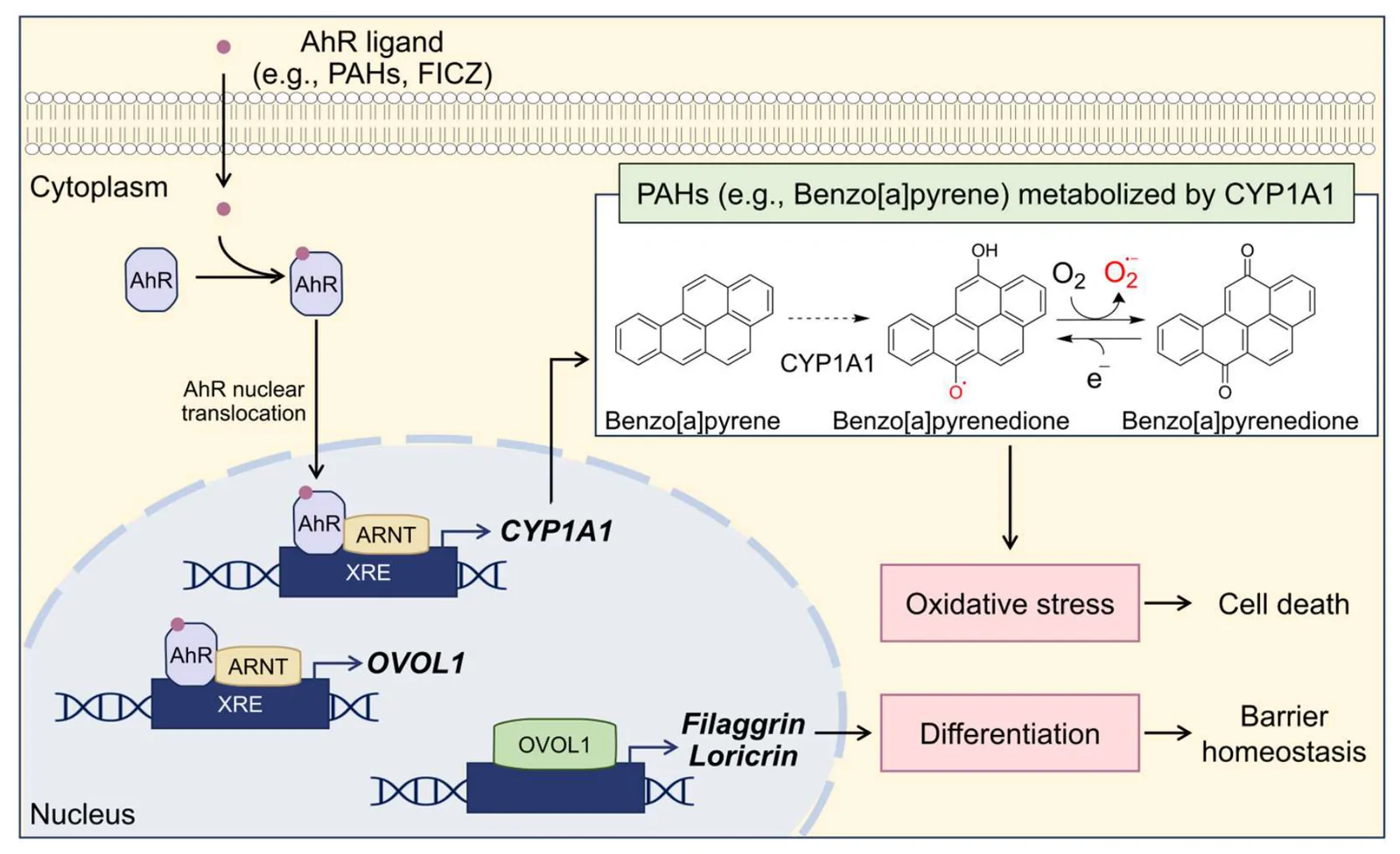
Dual roles of AhR signaling in pollutant toxicity and skin damage. AhR signaling exhibits a context-dependent dual role in the skin. Under physiological conditions, moderate AhR activation promotes epidermal differentiation and barrier maintenance through the AhR–OVOL1–filaggrin axis. In contrast, excessive activation by environmental pollutants such as PAHs enhances CYP1A1-mediated xenobiotic metabolism, leading to accumulation of reactive intermediates and oxidative stress, thereby contributing to epidermal damage.

In the context of environmental pollution, this ligand-dependent divergence is particularly relevant. Persistent exposure to PAHs present in PM can effectively sustain activation of the AhR signaling system, driving sustained CYP1A1 activity, oxidative stress generation, and inflammatory signaling. Consequently, although AhR plays essential physiological roles in maintaining epidermal barrier integrity, its activation by persistent environmental toxicants may contribute to pollution-associated skin damage (Figure 2).

### 2.5. The Keap1–Nrf2–ARE Pathway: Antioxidant Defense and Cytoprotection

To maintain cutaneous redox homeostasis, the skin relies on the Nrf2 signaling pathway. Nrf2 acts as a master transcriptional regulator that coordinates the expression of numerous cytoprotective genes to defend keratinocytes against PM-induced oxidative injury [125]. Under basal, unstressed conditions, intracellular Nrf2 protein levels remain low due to rapid proteasomal degradation. Nrf2 is a short-lived protein with a half-life of approximately 15–40 min, and about 15 min in HaCaT keratinocytes [154,155].

This strict negative regulation is mediated by Kelch-like ECH-associated protein 1 (Keap1), which contains several functional domains that precisely control Nrf2 stability [156]. The N-terminal Broad complex-Tramtrack-Bric-à-brac (BTB) domain mediates Keap1 homodimerization and recruits Cullin-3 (Cul3) to assemble the E3 ubiquitin ligase complex that targets Nrf2 for continuous ubiquitination and proteasomal degradation [60]. The central intervening region (IVR), containing cysteine residues such as Cys151, Cys273, and Cys288, functions as a sensor of electrophiles and oxidants. The C-terminal Kelch repeat domain directly interacts with the Neh2 regulatory domain of Nrf2 through the widely accepted “hinge-and-latch” mechanism [157]. In this model, Keap1 recognizes a high-affinity ETGE motif (the stable hinge) and a lower-affinity DLG motif (the dynamic latch). Under homeostatic conditions, this dual-binding configuration positions lysine residues on Nrf2 for efficient Cul3-mediated ubiquitination, thereby preventing unnecessary antioxidant activation.

Upon oxidative or electrophilic modification of Keap1 cysteine residues, or disruption of the Keap1–Nrf2 interaction by small-molecule protein–protein interaction (PPI) modulators, a conformational change weakens the interaction with the DLG motif while the ETGE hinge remains intact [158]. For example, LH601A is a direct Keap1–Nrf2 PPI inhibitor that targets the Keap1 Kelch domain and disrupts Nrf2 binding, thereby promoting Nrf2 activation [158]. This partial dissociation alters the spatial alignment of lysine residues between the ETGE and DLG motifs, thereby impairing the Cul3-mediated ubiquitination [159]. Consequently, newly synthesized Nrf2 escapes Keap1-dependent degradation, accumulates in the cytoplasm, and subsequently translocates to the nucleus. In the nucleus, Nrf2 heterodimerizes with small musculoaponeurotic fibrosarcoma (sMaf) transcription factors, and the Nrf2–sMaf complex binds antioxidant response elements (AREs) in the promoter regions of target genes. This transcriptional program induces a wide range of antioxidant and detoxifying enzymes that enhance cellular resistance to oxidative stress [53,160]. Once oxidative stress subsides, Keap1-dependent ubiquitination resumes, leading to Nrf2 degradation and termination of the transcriptional response [161].

Among Nrf2 downstream targets, heme oxygenase-1 (HO-1) and NAD(P)H-quinone oxidoreductase 1 (NQO1) are key components of the antioxidant defense system [162]. HO-1 degrades pro-oxidant heme into biliverdin, carbon monoxide, and free iron; biliverdin is subsequently converted to bilirubin, a potent endogenous antioxidant capable of scavenging ROS [163]. NQO1 protects cells by catalyzing the two-electron reduction in quinones, preventing redox cycling and limiting reactive oxygen species generation [164,165]. Additional Nrf2-regulated genes include enzymes involved in glutathione biosynthesis, such as glutamate–cysteine ligase (GCL), and detoxification enzymes, including glutathione S-transferases (GSTs), which collectively reinforce cellular antioxidant capacity [161]. Through coordinated induction of these cytoprotective genes, Nrf2 plays a central role in maintaining cellular redox equilibrium and protecting keratinocytes from oxidative damage.

Importantly, Keap1 modification in response to thiol-reactive stress follows a dose-dependent continuum rather than a linear relationship. This defines a therapeutic window in which moderate Nrf2 activation is cytoprotective, whereas insufficient or excessive activation may disrupt cellular homeostasis [154]. Consistent with this concept, Nrf2 signaling plays a context-dependent role in epidermal biology [166]. Both loss and constitutive activation of Nrf2 exacerbate disease severity in atopic dermatitis-like mouse models, likely due to impaired keratinocyte differentiation and barrier dysfunction. In contrast, controlled pharmacological activation moderately improves epidermal barrier function, highlighting the need for precise regulation of Nrf2 activation intensity and duration. However, endogenous Nrf2 activation alone may be insufficient to counteract the excessive ROS production induced by PM exposure [125], suggesting that controlled pharmacological enhancement of Nrf2 signaling may help restore protective Nrf2 activity and provide additional protection against PM-induced cell damage.

**Figure 3:**
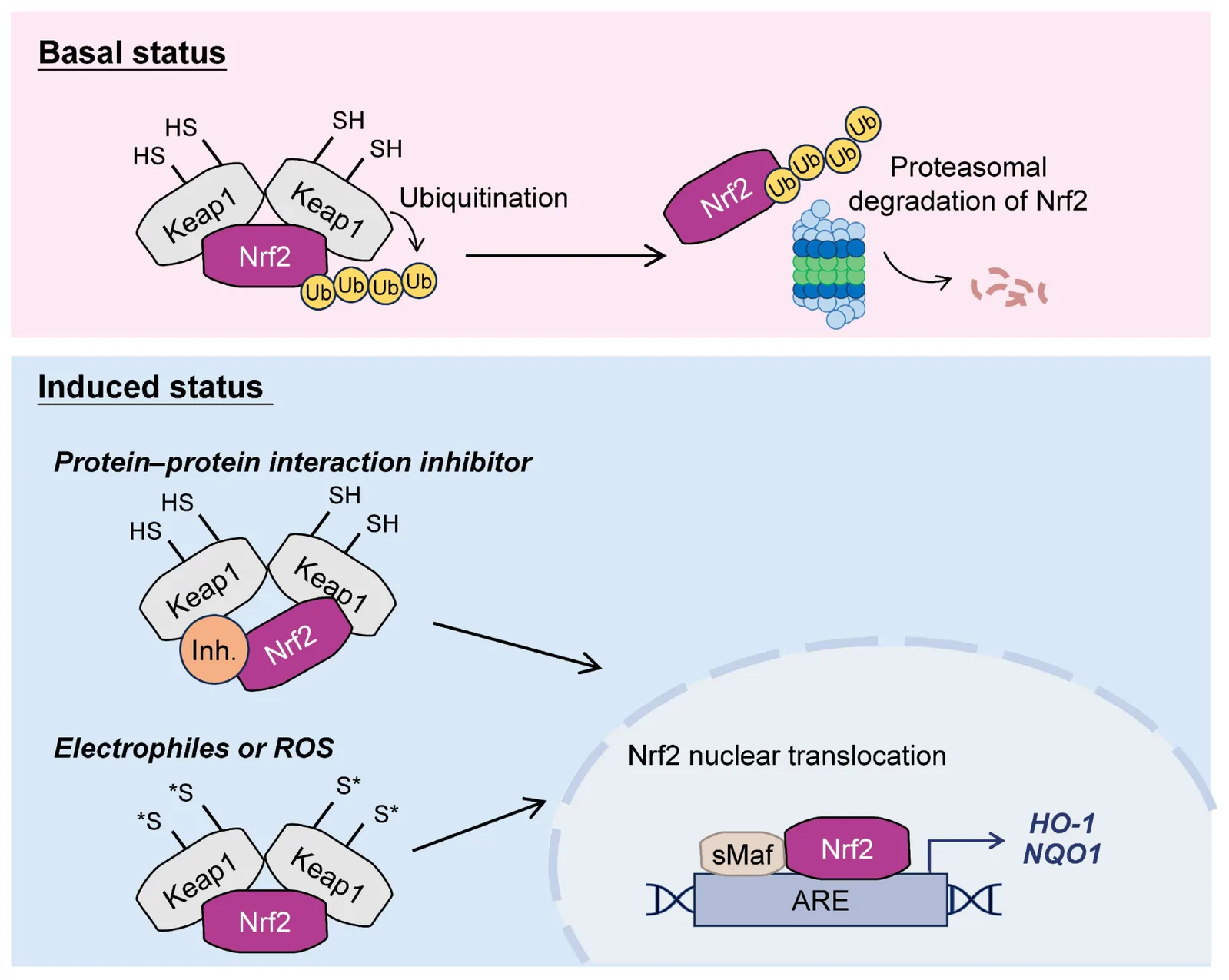
The Keap1–Nrf2 antioxidant defense pathway. The Keap1–Nrf2 signaling pathway regulates cellular antioxidant defense through distinct activation mechanisms. Under basal conditions, Nrf2 is bound to Keap1 in the cytoplasm and undergoes continuous ubiquitination and proteasomal degradation, maintaining low basal activity. Upon oxidative stress or exposure to electrophiles, reactive species modify critical cysteine residues on Keap1, leading to conformational changes that impair Nrf2 ubiquitination. As a result, stabilized Nrf2 accumulates and translocates into the nucleus. In addition, disruption of the Keap1–Nrf2 protein–protein interaction by small molecules prevents Nrf2 binding to Keap1, thereby blocking its degradation and promoting nuclear translocation. In both activation modes, nuclear Nrf2 binds to ARE and induces the expression of cytoprotective genes, including HO-1 and NQO1, thereby enhancing cellular defense against oxidative stress. Inh., inhibitor; SH, reduced cysteine; S*, modified cysteine.

Collectively, the Keap1–Nrf2–ARE signaling axis represents a central cytoprotective network that safeguards the skin against environmental oxidative stress (Figure 3). Emerging evidence further indicates functional crosstalk between the AhR and Nrf2 pathways. Beyond their downstream signaling interactions, AhR and Nrf2 have been proposed to participate in a shared transcriptional regulatory network involving the AhR–Nrf2 gene battery. This concept has been further extended by the identification of Jun dimerization protein 2 (JDP2) as a chromatin regulator involved in coordinating AhR- and Nrf2-dependent transcriptional responses [167]. In particular, AhR activation may partially contribute to activation of the Nrf2/HO-1 axis by binding to XREs within the Nrf2 gene locus [168,169,170]. Together with AhR-mediated pathways, Nrf2 constitutes a key regulatory node controlling cellular responses to PM exposure. Targeting these interconnected pathways may therefore restore redox homeostasis, reduce oxidative injury, and preserve epidermal barrier integrity, providing a mechanistic framework for developing protective interventions against pollution-associated skin damage.

## 3. Mechanism-Based Strategies Targeting AhR and Nrf2 Pathways

### 3.1. Nrf2-Dominant Antioxidant Strategies

Early therapeutic strategies targeting PM-induced skin injury primarily focused on mitigating oxidative stress [171,172]. In response, pharmacological activation of Nrf2 has emerged as a central strategy to restore redox homeostasis. Nrf2 activators can be broadly categorized based on their predominant mechanisms, including covalent modification of Keap1 cysteine residues, disruption of Keap1–Nrf2 protein–protein interactions, and activation of upstream kinase signaling pathways. However, it should be noted that many of these compounds also exhibit secondary effects on other stress-response pathways, including AhR signaling, depending on cellular context.

One major group consists of electrophilic or pro-electrophilic compounds such as artemisitene and sulforaphane that activate Nrf2 through covalent modification of Keap1 cysteine residues (especially Cys 151), thereby disrupting Keap1-mediated ubiquitination and stabilizing Nrf2 [173,174,175]. Artemisitene has been reported to alleviate bleomycin-induced lung injury and inflammation, consistent with its role in Nrf2-mediated cytoprotection [176]. Similarly, sulforaphane has been reported to reduce PM-induced ROS accumulation and oxidative stress in keratinocytes [103].

A second class consists of compounds that interfere with the interaction between the Keap1 Kelch domain and the Nrf2 DLG/ETGE motif. Capsaicin has been reported to disrupt this interaction and reduce oxidative injury in cellular stress models [177]. In addition, several natural flavonoids have been suggested to target the Keap1 Kelch domain and stabilize Nrf2. For example, tiliroside has been reported to bind to the Kelch domain of Keap1 and protect against acetaminophen (APAP)-induced acute liver injury in mice [178]. Similarly, epigallocatechin-3-gallate (EGCG) has been reported to promote Nrf2 nuclear translocation and enhance keratin 16 expression during wound repair, and molecular docking analyses suggest that EGCG may interact with the Keap1 Kelch domain to inhibit Keap1-mediated degradation of Nrf2 [179]. In a 2,4-dinitrochlorobenzene (DNCB)-induced atopic dermatitis mouse model, EGCG also mitigated PM-associated inflammatory responses and preserved keratinocyte differentiation markers, including loricrin and filaggrin [180]. Similarly, a flavonoid-rich fraction from *Phyla nodiflora* has been reported to activate the Nrf2/HO-1 axis and attenuate PM-induced oxidative stress in keratinocytes, potentially through promotion of Keap1 degradation and interaction with the Keap1 Kelch domain [135].

A third category includes upstream kinase regulators that activate Nrf2 indirectly through signaling cascades such as MAPK, AMP-activated protein kinase α (AMPKα), phosphoinositide 3-kinases (PI3K)/Akt, and protein kinase C (PKC) [181,182]. Activation of these pathways enhances Nrf2 phosphorylation and promotes its nuclear translocation. Several flavonoids have been reported to activate these signaling pathways. For instance, xanthohumol has been reported to stimulate AMPKα signaling and promote Nrf2 activation, thereby strengthening antioxidant defense systems in oxidative stress models [183].

Although antioxidant-based interventions effectively restore redox homeostasis in PM-exposed systems, their ability to fully repair epidermal barrier dysfunction remains limited. Oxidative stress represents only one component of PM-induced skin pathology, whereas structural deterioration of the epidermal barrier arises primarily from impaired keratinocyte differentiation and disruption of intercellular junctions. Therefore, strategies focused predominantly on antioxidant responses may not be sufficient to completely restore epidermal integrity. These observations suggest that additional regulatory pathways involved in epidermal differentiation and barrier formation may also need to be considered when developing therapeutic strategies against PM-induced skin damage.

Importantly, several compounds categorized within Nrf2-dominant strategies have also been reported to modulate AhR signaling. For example, capsaicin and EGCG can inhibit AhR nuclear translocation or transcriptional activity in a context-dependent manner [184,185], while xanthohumol has been reported to inhibit AhR activation under xenobiotic stress conditions [186]. These observations suggest that the functional boundaries between Nrf2- and AhR-regulated pathways are often blurred, supporting the concept that therapeutic strategies should be interpreted based on dominant pathway preference rather than strict mechanistic exclusivity.

### 3.2. AhR-Dominant Epidermal Regulatory Strategies

Given the central role of the AhR in sensing environmental toxicants and regulating barrier differentiation, pharmacological targeting of AhR has emerged as another potential strategy to mitigate PM-induced skin injury. However, therapeutic manipulation of AhR remains challenging due to its dual biological functions, often described as the “AhR paradox”. Two contrasting approaches have therefore been explored.

On one hand, inhibition of AhR signaling has been proposed as a strategy to prevent pollutant-induced toxicity. By blocking PAH binding to AhR, this approach suppresses downstream xenobiotic metabolism and reduces CYP1A1 induction. Several studies have explored this concept using AhR antagonists. For example, the monoterpenoid S-carvone acts as an allosteric inhibitor of AhR signaling and reduces UV- and benzo[a]pyrene-induced inflammatory responses in mouse skin [187]. Similarly, polyphenol-enriched supplements have been reported to attenuate AhR/ARNT-dependent inflammatory mediators such as COX-2 and MMP-1 induced by PM exposure [188]. While this strategy can reduce pollutant-driven oxidative response and inflammation, sustained AhR inhibition may also interfere with physiological pathways involved in epidermal differentiation, particularly the AhR–OVOL1–filaggrin axis required for barrier maintenance.

Conversely, controlled activation of AhR signaling has been explored as a strategy to promote epidermal differentiation and restore barrier function. Diosmin activates AhR signaling and upregulates epidermal differentiation markers such as filaggrin and loricrin through OVOL1 regulation [189]. Curcumin has also been reported to modulate AhR signaling while suppressing inflammatory responses, including NF-κB activation [190]. Hydroxymetabolites derived from vitamin D3, lumisterol, and tachysterol have been identified as endogenous regulators that can act through AhR and other nuclear receptors, promote keratinocyte differentiation, and enhance protection against oxidative and DNA damage [191,192]. In particular, CYP11A1- and CYP27A1-derived lumisterol metabolites have been shown to interact with AhR and regulate keratinocyte differentiation and cellular responses to oxidative stress [192]. Melatonin and its metabolites may represent another endogenous source of AhR modulation, as they have been reported to activate AhR and induce AhR nuclear translocation in human keratinocytes [193]. Together, these observations indicate that AhR activation contributes not only to xenobiotic sensing but also to epidermal homeostasis and barrier restoration. Notably, these compounds also concurrently influence Nrf2-associated antioxidant responses, highlighting pathway interdependence [194,195,196,197].

To address the potential risks associated with excessive AhR activation, recent studies have introduced the concept of selective AhR modulators (SAhRMs), which exhibit ligand-specific and context-dependent transcriptional outcomes [198,199]. Ligand binding induces distinct conformational changes in AhR, thereby influencing its interaction with cofactors, DNA response elements, and post-translational modifications, ultimately leading to cell- and tissue-specific gene expression profiles. These properties may underlie the differential induction of xenobiotic-metabolizing enzymes such as CYP1A1 and other downstream targets. Accordingly, current research highlights the importance of identifying AhR modulators that favor protective pathways, such as those involved in epidermal differentiation (e.g., filaggrin), while minimizing adverse metabolic responses [200].

Despite these advances, strategies targeting AhR signaling alone primarily regulate inflammatory responses and epithelial differentiation pathways but do not directly address the oxidative burden generated during pollutant exposure. Because PM-induced skin injury arises from both oxidative stress and structural barrier disruption, modulation of AhR signaling alone may be insufficient to fully counteract pollution-associated skin damage. These limitations highlight the need for therapeutic strategies capable of simultaneously regulating oxidative stress and epithelial homeostasis.

### 3.3. Emerging Perspectives: Coordinated Regulation of AhR and Nrf2

This concept is particularly relevant to PM-induced skin injury, in which oxidative stress and barrier dysfunction occur simultaneously and cannot be fully addressed by single-pathway intervention. The limitations of single-pathway interventions have led to increasing interest in therapeutic strategies capable of simultaneously regulating xenobiotic sensing and antioxidant defense mechanisms. Although these strategies are categorized according to their primary mechanistic emphasis, increasing evidence indicates that AhR and Nrf2 signaling are highly interconnected rather than functionally isolated.

Many electrophilic compounds that stabilize Nrf2 by covalently modifying Keap1 cysteine residues, particularly Cys151, may also influence AhR signaling. Representative examples include sulforaphane [201,202], dimethyl fumarate [203,204,205], and *tert*-butyl hydroquinone [204,206,207], all of which have been reported to exhibit context-dependent interactions with AhR signaling pathways. Notably, dimethyl fumarate, a clinically used treatment for psoriasis, is metabolized to monomethyl fumarate, which has been reported to exert direct antiproliferative, prodifferentiative, and anti-inflammatory effects on keratinocytes [208,209]. Likewise, several compounds initially characterized as AhR modulators, such as diosmin and curcumin, have been reported to affect oxidative stress responses through secondary activation of Nrf2-related pathways [189,190,194,195]. These studies collectively suggest that the distinction between Nrf2-dominant and AhR-dominant strategies is often not absolute, but rather reflects differences in dominant pathway preference. Therefore, increasing attention has been directed toward compounds that coordinately modulate both AhR and Nrf2 signaling networks.

Early approaches proposed combining AhR inhibition with Nrf2 activation to suppress CYP1A1-derived ROS while enhancing antioxidant capacity [210]. However, as discussed above, antagonizing AhR may interfere with physiological signaling pathways involved in epidermal differentiation. Consequently, coordinated activation of AhR and Nrf2 may represent a potential strategy for counteracting PM-induced skin damage.

Mechanistically, these two pathways perform complementary functions. AhR signaling regulates epidermal barrier integrity by activating the AhR–OVOL1–filaggrin regulatory axis and by modulating the expression of several junctional proteins, while also influencing inflammatory responses in epithelial tissues. In contrast, Nrf2 functions as the principal cellular defense system against oxidative stress, activating a battery of antioxidant and detoxifying enzymes. Importantly, crosstalk between these pathways further strengthens their cooperative effects. Mechanistically, recent studies have revealed that this transcriptional coordination involves the AhR–Nrf2–JDP2 gene battery, in which the chromatin regulator JDP2 forms transcriptional complexes with AhR–ARNT and Nrf2–sMAF, facilitating coordinated recruitment to XREs and AREs within the AhR promoter [167]. These findings suggest that AhR and Nrf2 signaling are not merely parallel pathways but are connected through shared transcriptional machinery. In addition, AhR activation has been reported to contribute to Nrf2 transcription through XREs located within the Nrf2 gene locus [168,169,170]. Meanwhile, Nrf2 upregulates phase II detoxifying enzymes such as NQO1, which catalyzes the two-electron reduction in reactive quinones generated during CYP1A1-mediated metabolism [164,165]. Interestingly, NQO1 expression can also be influenced by AhR signaling, highlighting a shared cytoprotective node between these pathways. Through this coordinated mechanism, Nrf2-mediated antioxidant responses help mitigate the oxidative stress associated with AhR-driven xenobiotic metabolism.

**Figure 4:**
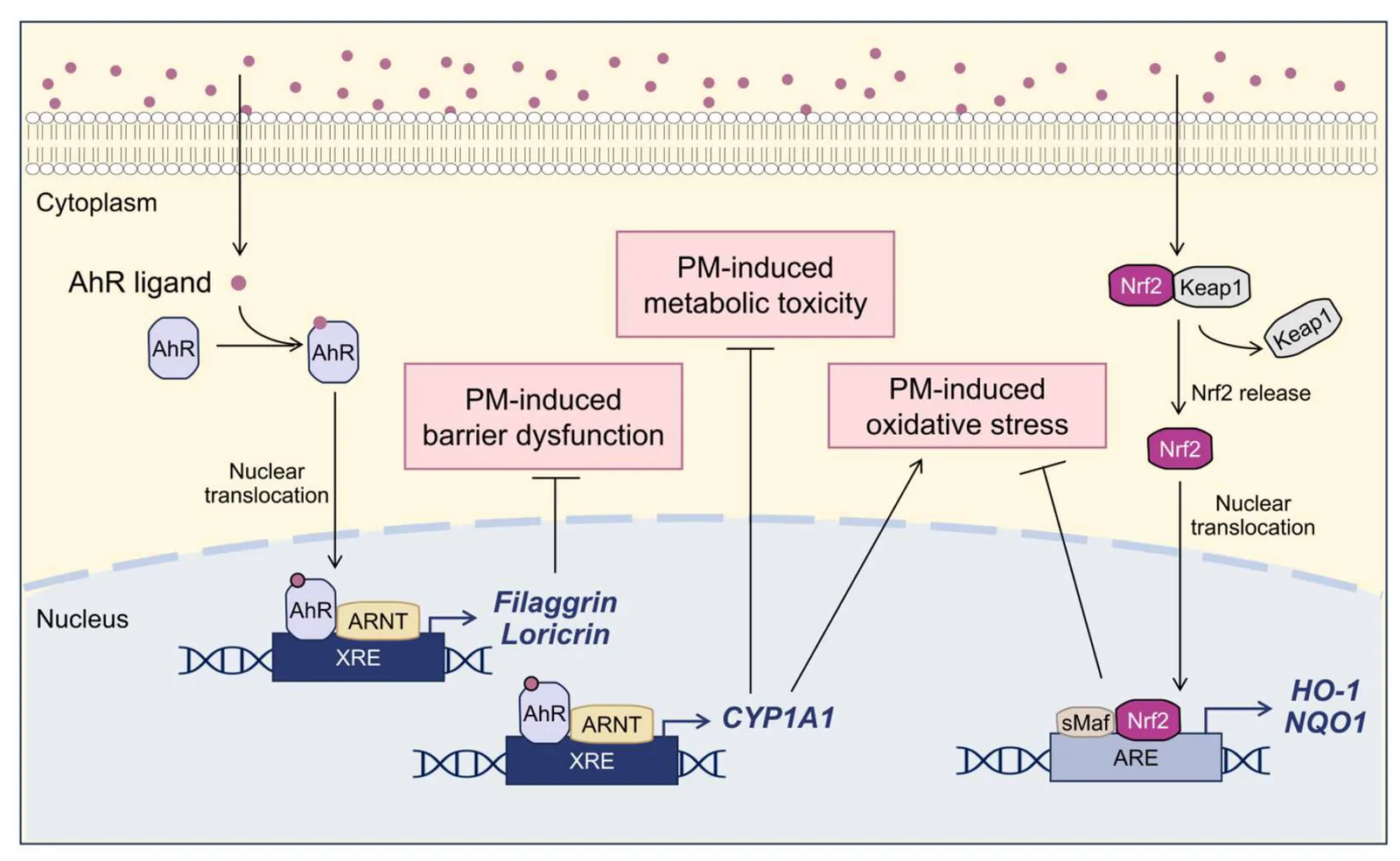
Conceptual framework of AhR–Nrf2 dual protection against PM-induced epidermal damage. PM induces epidermal injury through three interconnected axes: xenobiotic metabolism, oxidative stress, and barrier dysfunction. Dual activation of AhR and Nrf2 provides coordinated protection by regulating xenobiotic metabolism and promoting epidermal differentiation and barrier integrity via AhR signaling, while simultaneously enhancing antioxidant defense through Nrf2 activation. This integrated mechanism reduces oxidative burden and maintains epidermal homeostasis under PM exposure.

Collectively, dual activation of AhR and Nrf2 may overcome the limitations associated with single-pathway modulation. While AhR activation alone enhances xenobiotic metabolism, it may concurrently increase oxidative burden through CYP1A1 induction. Nrf2 activation alone primarily strengthens antioxidant defenses without directly regulating xenobiotic sensing or epidermal differentiation. Therefore, coordinated activation of both pathways enables a more balanced response, integrating enhanced detoxification capacity with effective antioxidant protection, ultimately reducing oxidative stress while preserving epidermal homeostasis (Figure 4).

Consistent with this concept, accumulating evidence suggests that certain natural compounds simultaneously activate both pathways and confer protective effects across epithelial systems. Prenylated xanthones from mangosteen (*Garcinia mangostana*) have been reported to co-activate AhR and Nrf2, thereby contributing to protection of intestinal barrier integrity [146]. Similarly, cannabidiol promotes epidermal differentiation and redox balance through AhR-OVOL1-filaggrin and AhR-Nrf2-NQO1 axes [211], while α-tocopherylquinone enhances tight junction formation by modulating claudin expression through both pathways [212]. In addition, environmental stimuli can also trigger coordinated activation of these pathways. For example, *Alternaria alternata* mycotoxins have been reported to induce nuclear translocation of both AhR and Nrf2 in colon epithelial cells, further illustrating the intrinsic crosstalk between xenobiotic sensing and antioxidant defense systems during epithelial stress responses [213]. Tapinarof is a representative therapeutic example of dual AhR–Nrf2 modulation and has been clinically approved for the treatment of plaque psoriasis and atopic dermatitis. Supported by preclinical models and clinical studies, tapinarof acts as an AhR agonist and promotes Nrf2-associated antioxidant responses. Through these mechanisms, tapinarof enhances epidermal differentiation, restores barrier function, and reduces inflammatory responses in skin disorders [214,215]. Representative compounds reported to modulate AhR and Nrf2 signaling across epithelial injury models are summarized in Table 2. Collectively, the available evidence supports the concept that coordinated modulation of AhR-mediated barrier regulation and Nrf2-dependent antioxidant defense as a promising therapeutic strategy for mitigating PM-induced skin barrier dysfunction.

## 4. Conceptual Integration of AhR–Nrf2 Interplay in PM-Induced Skin Injury

The interplay between AhR and Nrf2 signaling can be conceptualized as a coordinated regulatory system governing the epidermal response to PM exposure. Although their functions partially overlap, their activation is not necessarily coupled, reflecting ligand-specific and context-dependent transcriptional regulation.

Nrf2 primarily maintains redox homeostasis through antioxidant and cytoprotective gene induction, whereas AhR mediates xenobiotic sensing and regulates epidermal differentiation and barrier organization. Because PM-induced skin injury involves both oxidative stress and structural disruption, effective protection requires coordinated engagement of both pathways. Comparative observations further support the functional complementarity between AhR and Nrf2. Nrf2-dominant activation, exemplified by a flavonoid-rich fraction derived from *Phyla nodiflora*, attenuates PM-induced oxidative stress and partially restores hydration-related function through AQP3 regulation [135]. However, overall recovery of cellular damage remains incomplete, indicating that redox correction alone is insufficient to fully counteract PM-induced injury, likely due to the lack of direct engagement of AhR-dependent differentiation pathways. In contrast, dual-pathway modulation using tapinarof, a pharmacological AhR agonist with additional Nrf2-activatingcapacity, enables coordinated improvement of PM-induced oxidative stress, cell viability, and barrier-associated functions. This integrated response is associated with more effective restoration of epidermal homeostasis under PM-induced stress conditions [32], highlighting the functional advantage of simultaneous pathway engagement.

Collectively, the available evidence supports a conceptual model in which the balance and integration of AhR-mediated xenobiotic sensing and Nrf2-dependent antioxidant defense determine the overall outcome of PM exposure in the skin. In this model, Nrf2-driven responses primarily buffer oxidative stress, whereas AhR signaling supports xenobiotic metabolism and barrier organization. Coordinated activation of both pathways enables integration of detoxification, antioxidant protection, and structural maintenance, thereby enabling a more comprehensive adaptive response to environmental insults.

## 5. Conclusions and Perspectives

PM-induced skin damage arises from the convergence of oxidative stress and disruption of epidermal barrier integrity, highlighting the limitations of single-pathway strategies. Modulation of either antioxidant defense or barrier regulation alone is insufficient to fully restore epidermal function. Comparative evidence indicates that Nrf2-dominant activation primarily mitigates oxidative stress, whereas dual activation of AhR and Nrf2 enables coordinated regulation of redox homeostasis, xenobiotic metabolism, and barrier-associated processes. This distinction underscores the functional complementarity of these pathways. Taken together, the coordinated AhR and Nrf2 signaling represents a central regulatory node linking xenobiotic sensing, antioxidant defense, and epidermal homeostasis, providing a mechanistic basis for dual-targeted therapeutic strategies against PM-induced skin barrier dysfunction.

Despite growing evidence supporting the protective potential of coordinated AhR and Nrf2 activation, important knowledge gaps remain in the current understanding of dual-pathway regulation in PM-induced skin injury. Most studies are still based on in vitro systems, with limited integration of in vivo models that capture the complexity of physiological exposure conditions. In particular, how AhR–Nrf2 crosstalk is dynamically regulated under different PM exposure contexts, including variation in exposure duration, dose, and environmental composition, remains poorly understood. Future studies using advanced skin models and in vivo exposure systems will be essential to elucidate the context-dependent regulation of AhR and Nrf2 signaling under physiologically relevant conditions.

An additional unresolved issue concerns the translational generalizability of dual-pathway modulation. Although tapinarof exhibits coordinated modulation of AhR and Nrf2 signaling, it remains unclear whether such activity represents a compound-specific property or a broader class effect across different modulators. The impact of distinct activation profiles on adaptive responses under chronic PM exposure remains incompletely defined. Addressing this gap will require systematic comparative studies of structurally and mechanistically diverse AhR and Nrf2 modulators to establish the robustness of coordinated dual-pathway modulation strategies. Such studies will be essential for defining the translational potential of dual-pathway modulation in environmentally induced skin disorders.

From a translational perspective, future therapeutic development should focus on identifying selective modulators capable of coordinately enhancing epidermal differentiation, barrier integrity, and antioxidant capacity without triggering excessive xenobiotic toxicity. Such approaches may provide a foundation for next-generation topical strategies targeting environmentally induced skin disorders.

## Figures and Tables

**Table 2:** Representative AhR- and/or Nrf2-targeting natural compounds for protection against oxidative stress-induced barrier dysfunction.

Compound	Model	Mechanism	Molecular Effects	Outcomes	References
**Nrf2-dominant strategy**					
Capsaicin (capsaicinoid)	Ethanol-induced oxidative injury in human gastric mucosal epithelial cells (GES-1)	Covalent modification of Keap1 cysteine residues	Enhances Nrf2 nuclear translocation Upregulates HO-1, Trx, GSS and NQO1	Preserves mitochondrial function and alleviates oxidative mucosal injury	[177]
EGCG (catechin)	High-fat, high-fructose diet-induced metabolic stress in C57BL/6 mice	Disruption of Keap1–Nrf2 interaction (supported by docking analysis)	Enhances Nrf2 nuclear translocation Upregulates keratin 16	Accelerates re-epithelialization and wound healing	[179]
Xanthohumol (prenylated flavonoid)	H_2_O_2_- and high glucose-induced oxidative injury in human keratinocytes (HaCaT) Streptozotocin-induced diabetes in Sprague-Dawley rats	Activation of AMPKα–Nrf2 signaling Covalent modification of Keap1 cysteine residues	Enhances Nrf2 nuclear translocation Upregulates HO-1, NQO1, SOD1, and TrxR1	Reduces oxidative stress and promotes diabetic wound healing	[183]
**AhR-dominant strategy**					
S-Carvone (monoterpenoid)	UV- and/or BaP-induced skin inflammation in C57BL/6 mice	Noncompetitive AhR antagonism	Suppresses CYP1A1, CYP1A2, AhRR, and PAI-1 expression	Reduces UV- and BaP-induced inflammatory responses	[187]
Diosmin (flavone glycoside)	Th2 cytokine-induced barrier dysfunction in normal human epidermal keratinocytes	AhR agonism	Upregulates OVOL1, FLG, LOR, IVL, HRNR, and NQO1	Promotes keratinocyte differentiation and improves epidermal barrier integrity	[189]
FICZ (carbazole)	Sodium dodecyl sulfate-induced atopic dermatitis in NC/Nga mice	AhR agonism	Induces AhR nuclear translocation and CYP1A1 expression Restores FLG expression Suppresses IL-22 and IFN-γ expression	Reduces epidermal and dermal thickness and improves inflammation and TEWL	[148]
**AhR and Nrf2 dual-targeting strategy**					
Altertoxin II (mycotoxin)	IL-1β-induced inflammation in noncancerous colonic epithelial cells (HCEC-1CT)	Dual activation of AhR and Nrf2	Induces nuclear translocation of AhR and Nrf2 Suppresses NF-κB/p65 translocation and MMP-2 expression Restores claudin-4, ZO-1, and integrin β1 localization	Maintains epithelial barrier integrity and modulates inflammatory responses	[213]
Cannabidiol (non-psychoactive cannabinoid)	*tert*-butyl hydroperoxide-induced oxidative stress in normal human epidermal keratinocytes	Dual activation of AhR and Nrf2 (AhR–Nrf2 axis)	Enhances AhR transcriptional activity and nuclear translocation Promotes Nrf2 nuclear translocation Upregulates CYP1A1, AhRR, OVOL1, FLG, IVL, HO-1, and NQO1	Improves epidermal differentiation and redox balance	[211]
Cinnamaldehyde (phenylpropanoid)	BaP-induced oxidative stress in human keratinocytes (HaCaT)	AhR inhibition + Nrf2 activation	Inhibits AhR nuclear translocation and CYP1A1 expression Enhances Nrf2 nuclear translocation and HO-1 expression	Protects keratinocytes from oxidative stress	[210]
Garcinone D (prenylated xanthones)	*tert*-butyl hydroperoxide-induced ROS production in human intestinal cells (HT-29)	Dual activation of AhR and Nrf2	Enhances nuclear translocation of AhR and Nrf2 Upregulates CYP1A1 and HO-1 Restores tight junction proteins (ZO-1, occludin, claudin-1)	Improves barrier integrity and reduces oxidative stress	[146]
Tapinarof (stilbene)	Imiquimod-induced psoriasis-like dermatitis in Balb/c and C57Bl/6 mice	AhR agonism Dual activation of AhR and Nrf2	Upregulates CYP1A1, NQO1, FLG, IVL, and HRNR	Enhances epidermal differentiation and reduces skin inflammation	[32,215]
α-Tocopherylquinone (quinone)	Dextran sodium sulfate-induced colitis in C57BL/6J mice	Dual activation of AhR and Nrf2	Upregulates claudin-3 Activates AhR-XRE and Nrf2-ARE	Improves TER and attenuates intestinal inflammation	[212]

Abbreviation: AhR, aryl hydrocarbon receptor; AhRR, AhR repressor; AMPKα, AMP-activated protein kinase α; ARE, antioxidant response elements; BaP, benzo[a]pyrene; CYP1A1, cytochrome P450 1A1; FLG, filaggrin; GSS, glutathione synthetase; HO-1, heme oxygenase 1; HRNR, hornerin; IFN-γ, interferon-γ; IL-22, interleukin-22; IVL, involucrin; Keap1, Kelch-like ECH-associated protein 1; LOR, loricrin; MMP-2, matrix metalloproteinases-2; NF-κB, nuclear factor kappa-light-chain-enhancer of activated B cells; NQO1, NAD(P)H-quinone oxidoreductase 1; Nrf2, nuclear factor erythroid 2-related factor 2; OVOL1, ovo-like 1; PAI-1, plasminogen activator inhibitor-1; SOD1, superoxide dismutase-1; TER, transepithelial electrical resistance; TEWL, transepidermal water loss; Trx, thioredoxin; TrxR, thioredoxin reductase; UV, ultraviolet; XRE, xenobiotic response element; ZO-1, zonula occludens-1.

## Data Availability

No new data were created or analyzed in this study. Data sharing is not applicable to this article.

## Abbreviations

The following abbreviations are used in this manuscript:

AhR	aryl hydrocarbon receptor
AhRR	AhR repressor
AIP	AhR-interacting protein
AMPKα	AMP-activated protein kinase α
AP-1	activator protein-1
APAP	acetaminophen
AQP3	aquaporin-3
AREs	antioxidant response elements
ARNT	AhR nuclear translocator
BTB	Broad complex-Tramtrack-Bric-à-brac
COX-2	cyclooxygenase-2
Cul3	Cullin-3
CYP1A1	cytochrome P450 1A1
DHE	dihydroethidium
ECL	enhanced chemiluminescence
EGCG	epigallocatechin-3-gallate
FBMN	feature-based molecular networking
FDA	U.S. Food and Drug Administration
FICZ	6-formylindolo [3,2-*b*]carbazole
FLG	filaggrin
GCL	glutamate–cysteine ligase
GCLC	glutamate–cysteine ligase catalytic subunit
GSH	glutathione
GSH-Px	glutathione peroxidase
GSS	glutathione synthetase
GSTs	glutathione S-transferases
γH2AX	phosphorylated histone H2AX
H_2_DCFDA	2′,7′-dichlorodihydrofluorescein diacetate
4-HNE	4-hydroxynonenal
HO-1	heme oxygenase 1
HRNR	hornerin
HSP90	90 kDa heat shock protein
IFN-γ	interferon-γ
IL-1β	interleukin-1β
iNOS	inducible nitric oxide synthase
IVL	involucrin
IVR	intervening region
JDP2	Jun dimerization protein 2
Keap1	Kelch-like ECH-associated protein 1
LC3-I	cytosolic form of microtubule-associated protein 1 light chain 3
LC3-II	lipidated form of microtubule-associated protein 1 light chain 3
LDH	lactate dehydrogenase
LOR	loricrin
Δψm	mitochondrial membrane potential
MAPKs	mitogen-activated protein kinase
MDA	malondialdehyde
MITF	melanocyte inducing transcription factor
MMPs	matrix metalloproteinases
NF-κB	nuclear factor kappa-light-chain-enhancer of activated B cells
NLRP1	NLR family pyrin domain containing 1
NMF	natural moisturizing factor
NOX4	NADPH oxidase 4
NQO1	NAD(P)H-quinone oxidoreductase 1
Nrf2	nuclear factor erythroid 2–related factor 2
OVOL1	ovo-like 1
8-oxo-dG	8-oxo-2′-deoxyguanosine
PAHs	polycyclic aromatic hydrocarbons
PAI-1	plasminogen activator inhibitor-1
PARP	poly(ADP-ribose) polymerase
PI3K	phosphoinositide 3-kinases
PKC	protein kinase C
PM	particulate matter
PPI	protein–protein interaction
ROS	reactive oxygen species
SAhRMs	selective AhR modulators
SASP	senescence-associated secretory phenotype
SA-β-gal	senescence-associated β-galactosidase
sMaf	small musculoaponeurotic fibrosarcoma
SOD	superoxide dismutase
Tapinarof	3,5-dihydroxy-4-isopropylstilbene
TBHQ	*tert*-butyl hydroquinone
TCDD	2,3,7,8-tetrachlorodibenzo-*p*-dioxin
TER	transepithelial electrical resistance
TEWL	transepidermal water loss
TLR5	toll-like receptor 5
TNF-α	tumor necrosis factor-α
TRP-1	tyrosinase-related protein 1
Trx	thioredoxin
TrxR	thioredoxin reductase
TYR	tyrosinase
UFPs	ultrafine particles
USEPA	United States Environmental Protection Agency
UV	ultraviolet
WHO	World Health Organization
XREs	xenobiotic response elements
ZO-1	zonula occludens-1
